# The Metropolitan Asylums Board and Sick Children: Inhumanity or Progress?

**Published:** 1913-09-27

**Authors:** 


### The Metropolitan Asylums Board and Sick Children: Inhumanity or Progress?

*To the Editor of* The Hospital.

Sir,—The discussion which has taken place recently in your columns concerning the new order in respect of the surgical treatment of sick children in Queen Mary's Hospital for Children, Carshalton, and in other institutions controlled by the Asylums Board, has been exceedingly valuable to all who follow the evolution of the twentieth-century awakening to the welfare of the nation's children. After Dr. Parson's able letter in your issue of September 13, I do not propose to repeat the substance of his arguments, with which I find myself in agreement. But inasmuch as the letter has not drawn any answer from the upholders of the other point of view, it does seem worth while to insist upon the necessity of displaying the utmost tenderness to the sentiments of the parents, even if those sentiments are in most cases deeply tinged by a somewhat exaggerated sentimentality. Anyone who has had charge of hospital wards containing children knows the extraordinary medley of good intentions and indifferent performances which make up the conduct of the average working-class parents to their offspring. And the amount of tact required to surmount the objections of this class to necessary and salutary measures of medical and surgical treatment is often very great. However, troublesome though the expenditure of the necessary time and patience may be, the result is worth the bother; and to induce anxious parents to be reasonable by soft words is better than to attempt to ride rough-shod over their peculiar ideas and prejudices. The question of distance from home, particularly when operative surgery has to be faced, is a very real stumbling-block to patients of this sort, or rather to their parents; and I think Dr. Morgan underrates it in his letter of September 6. For this reason beyond all others I support Dr. Parson's contentions, and shall continue to do so unless more cogent arguments are advanced against them than I have so far seen.—Yours faithfully,

M.D.
(formerly Great Ormond Street).

